# Exploring the link between chromosomal polymorphisms and reproductive abnormalities

**DOI:** 10.1186/s12978-024-01854-6

**Published:** 2024-09-05

**Authors:** Haiyan Pang, Tong Zhang, Xin Yi, Xiaojing Cheng, Guiling Wang

**Affiliations:** Department of Reproductive Medicine, Affiliated Hospital of Shandong Second Medical University, Weifang, Shandong China

**Keywords:** Chromosomal polymorphisms, Karyotyping, Infertility, Fetal anomalies

## Abstract

**Objective:**

This work aimed to investigate the potential correlation between chromosomal polymorphisms and various reproductive abnormalities.

**Methods:**

We examined 21,916 patients affected by infertility who sought care at the Department of Reproductive Medicine, Affiliated Hospital of Shandong Second Medical University between January 2018 and December 2022. A total of 2227 individuals identified as chromosomal polymorphism carriers constituted the polymorphism group, and 2245 individuals with normal chromosome karyotypes were randomly selected to form a control group. Clinical manifestations, histories of spontaneous miscarriage, abnormal reproductive developments, fetal abnormalities, and male sperm quality anomalies were statistically compared between these two groups.

**Results:**

Of the 21,916 patients analyzed, 2227 displayed chromosomal polymorphism, representing a 10.16% detection rate. Amongst the male patients, 1622 out of 10,827 exhibited polymorphisms (14.98%), whereas 605 out of 11,089 females showed polymorphisms (5.46%). Female carriers in the polymorphism group, showed statistically significant increased rates of spontaneous abortion (29.75% vs. 18.54%), fetal anomalies (1.32% vs. 0.81%), and uterine abnormalities compared with the control group (1.32% vs. 0.81%). Male carriers in the polymorphism group had higher rates of spontaneous abortion in partners (22.87% vs. 10.37%), fetal anomalies (1.97% vs. 0.25%), compromised sperm quality (41.74% vs. 7.18%), testicular underdevelopment (2.28% vs. 0.92%), and hypogonadotropic hypogonadism (0.62% vs. 0.37%) compared with the control group.

**Conclusion:**

Chromosomal polymorphisms may have a certain negative effect on reproductive irregularities, including spontaneous abortions, fetal anomalies, and reduced sperm quality in males. Their clinical effects deserve further investigation.

## Introduction

Infertility affects 25% of reproductive-aged couples in China. Chromosome examination is one of the important items in the clinical examination of infertile couples. Chromosomes serve as the vital carriers of genetic material. Chromosomal abnormalities often underpin complications such as infertility, miscarriage, stillbirths, birth defects, and other negative pregnancy outcomes [1, 2]. With advancements in chromosome banding technology, intricate observations of chromosome morphology and structure have become possible. This development has led to the identification of minute, consistent variations in chromosome structure, banding width, and staining intensity that are inherited in a Mendelian fashion. These variations are referred to as chromosomal polymorphisms. Such polymorphic variations predominantly occur in heterochromatin regions, composed mostly of non-coding, highly repetitive sequences that lack structural genes. Traditionally, these sequences are believed to neither possess transcriptional activity nor induce phenotypic effects [3, 4].

However, recent studies posit that variants in heterochromatin regions can instigate reproductive anomalies such as infertility and miscarriage [5]. Mechanisms proposed include gene position effects, epigenetic modifications, and disruptions in meiosis [6]. Infertility and miscarriage are caused by various complex factors, but genetic factors are one of the important causes of miscarriage. Clinically, correlations have been observed between chromosomal polymorphisms and a range of conditions: recurrent spontaneous abortions [7], male fertility issues including diminished sperm quality [8], and genital development abnormalities in both genders(Cavalcante et al. 2023). The relationship between chromosomal polymorphisms and pregnancy outcomes remains controversial. Our research aims to enhance the understanding of the influence of chromosomal polymorphisms on adverse pregnancy outcomes.

## Methods

### Study population

We enrolled 21,916 patients (10,827 males and 11,089 females) who visited the Department of Reproductive Medicine, Affiliated Hospital of Shandong Second Medical University between January 2018 and December 2022. Patients ranged in age from 22 to 43 years. From this population, we identified carriers of chromosomal polymorphisms (with normal karyotypes in their spouses) for our polymorphism group. Simultaneously, 2245 individuals with normal karyotypes (and normal spousal karyotypes) were randomly selected from the initial 21,916 as a control group, comprising 615 female and 1630 male controls. We found no significant difference in age between patients in the chromosomal polymorphism group and the control group. All outpatients with incomplete clinical data or those who had other systemic diseases were not included. All outpatients followed the IVF-ET protocols in our center. The study received approval from the Ethics Committee (Ethics Code. wyfy-2023-ky-154) of the Affiliated Hospital of Shandong Second Medical University.

### Karyotyping methodology

For chromosomal analysis, 2 mL of peripheral blood was drawn and anticoagulated with heparin. This step was followed by a culture period lasting between 68 and 72 h. Subsequently, lymphocytes were harvested and processed using standard methods. Where required, G-banding and C-banding techniques were employed. For each patient, 30 metaphases exhibiting optimal dispersion were counted, and between 5 and 10 karyotypes were analyzed. In instances of abnormalities, the count and analysis were repeated. Result interpretation adhered to the International System for the Nomenclature of Human Cytogenetics (ISCN 2020) guidelines. Any chromosomal irregularities were rigorously reviewed by a minimum of two geneticists.

### Semen analysis

Semen samples of all participating patients were collected into a sterile plastic specimen tube from a male partner who had been abstinent for 2–7 days. Semen samples underwent liquefaction, measurement of volume, sperm concentration, and motility, and morphological evaluation under an optical microscope in sequence according to manual Papanicolaou sperm staining. The WHO reference values for human semen characteristics were adopted as the reference values for normal semen quality [10]. Azoospermia was defined as the total absence of sperm cells in seminal liquid. Oligospermia was defined as a sperm concentration < 15 million/mL. Asthenozoospermia was defined as progressive motility (%) < 32%. Mild asthenozoospermia was defined as progressive motility (%) 20%−32%. Moderate asthenozoospermia was defined as progressive motility (%) 10%−20%. Severe asthenozoospermia was defined as progressive motility (%)1%−10%. Teratozoospermia was defined as morphologically normal forms (%) < 4%. Mild teratozoospermia was defined as morphologically normal forms (%) < 3%. Moderate teratozoospermia was defined as morphologically normal forms (%) < 2%. Severe teratozoospermia was defined as morphologically normal forms (%) < 1%. The normal semen group included men with a sperm concentration ≥ 15 million/mL, progressive motility (%) ≥ 32%, and morphologically normal forms (%) ≥ 4%.

### Ultrasound examination and follow-up

All pregnant women were diagnosed through 3D and 4D ultrasound examinations. Color Doppler ultrasound examination was as follows: The GE voluson E10 color Doppler ultrasound examination instrument was used, with a 3D volume probe frequency of 4.0–8.5 MHz and a 4D volume probe frequency of 2.5–7.0 MHz. The system was equipped with a workstation that can automatically store and collect ultrasound images. The specific operation was as follows. The pregnant woman was instructed to take a lateral or supine position, and the abdomen was fully exposed, Firstly, 2D exploration was conducted, during which observe the umbilical cord, umbilical artery, placenta, bladder, kidney, liver, gallbladder, gastrointestinal, diaphragmatic, lung, heart, chest, limbs, spine, neck, facial, cranial and amniotic fluid of the fetus were observed. Secondly, detailed exploration of the fetal growth and development from multiple angles and sections. For pregnant women suspected of fetal malformations, a 3D/4D ultrasound examination was performed until clear images of fetal growth were observed and stored. If the diagnosis could not be made at once, the pregnant woman was allowed to move or adjust her posture until the image was satisfactory before storage. For those with severe malformations detected, further treatment will be made after considering ethics and the wishes of the fetal parents. Those who continued to conceive were followed up until 6 months after the birth of the fetus.

### Statistical analysis

Data analysis was conducted using SPSS version 22.0. Statistical significance was set at P < 0.05. For numerical variables, the homogeneity of variance was checked by Levene’s test, and data normality was determined using the Kolmogorov–Smirnov test. Continuous variables are presented as mean ± standard errors and compared between groups using one-way ANOVA, Mann–Whitney U test, or Kruskal–Wallis test, as appropriate. Pearson’s chi-square or Fisher’s exact tests were used to compare categorical variables. amongst groups.

## Results

### Detection rate of chromosomal polymorphisms in the infertile population (Table 1)

**Table 1:** Detection rate of chromosomal polymorphisms in the infertile population

Chromosome polymorphism type	Chromosome karyotype	Frequency of polymorphism (n = 21,916)	Polymorphism frequency %
Y-chromosome polymorphisms		1082	4.94
	Yqh +	555	2.53
	Yqh−	526	2.40
	invY	1	0.0046
1, 9, 16 constrictions		628	2.87
	1qh +	344	1.57
	9qh +	139	0.63
	16qh +	134	0.61
	qh−	11	0.041
Group D/G anomalies		250	1.14
	Cenh +	38	0.17
	pstk +	152	0.69
	pss	27	0.12
	ps−	18	0.08
	ps +	15	0.07
Chromosome 9 inter-arm inversion	inv(9)	168	0.77
Complex polymorphisms		99	0.45
Total		2227	10.16

A total of 2227 carriers of chromosomal polymorphism were identified among 21,916 patients, yielding a detection rate of 10.16%. Within this cohort, 1622 male patients out of 10,827 were carriers, showing a detection rate of 14.98%, whereas 605 female patients out of 11,089 were carriers, resulting in a detection rate of 5.46%. The polymorphic variants identified were primarily categorized into five types: (1) Y chromosome polymorphisms: 1082 cases, constituting 48.59% of the polymorphisms, with a detection rate of 4.94%. It included 555 cases of 46, X, Y qh + , 526 cases of 46, X, Y qh−, and 1 case of 46, X, inv (Y) (p11.3q12) chromosome inversion. (2) Alterations in secondary constriction (encompassing chromosomes 1, 9, and 16): 628 cases, making up 28.20% of the polymorphisms, with a detection rate of 2.87%. This group included 617 cases of qh + (lengthening of secondary constriction) and 11 cases of qh− (shortening of secondary constriction). (3) Polymorphisms in D/G group satellites, stalks, and short arms: 250 cases, representing 11.23% of the polymorphisms, with a detection rate of 1.14%. This group included 152 cases of pstk + (stalk lengthening), 38 cases of cenh + (heterochromatin lengthening), 27 cases of pss (double satellite), 18 cases of ps− (satellite shortening), and 15 cases of ps + (satellite lengthening). (4) inv9 (inter-arm inversion of chromosome 9): 168 cases, accounting for 7.54% of the polymorphisms, with a detection rate of 0.77%. (5) Compound polymorphisms (presence of two or more polymorphic alterations in an individual): 99 cases, forming 4.45% of the polymorphisms, with a detection rate of 0.45%. The polymorphism type detected at the highest rate was Y chromosome polymorphism (4.94%), followed by sub-constrictive alterations in chromosomes 1, 9, and 16 (2.87%).

### Distribution of chromosomal polymorphisms ( Table 2)

**Table 2:** Distribution of chromosomal polymorphisms in infertile populations of different sexes

Chromosome type	Chromosome karyotype	Female polymorphism number/case	Female polymorphism frequency %	Male polymorphismnumber/case	Male polymorphism frequency %
Y chromosome polymorphism		0	0	1082	4.94
	Yqh +	0	0	555	2.53
	Yqh−	0	0	526	2.40
	invY	0	0	1	0.0046
1, 9, 16 constrictions		333	1.52	295	1.35
	1qh +	179	0.82	165	0.75
	9qh +	76	0.35	63	0.29
	16qh +	75	0.34	59	0.27
	qh−	3	0.014	8	0.037
Group D/G anomalies		151	0.69	99	0.45
	pstk +	96	0.44	56	0.26
	Cenh +	25	0.11	13	0.06
	pss	13	0.06	14	0.06
	ps−	9	0.04	9	0.04
	ps +	8	0.04	7	0.03
Chromosome 9 inter-arm inversion	Inv(9)	82	0.37	86	0.39
Complex polymorphisms	complex	39	0.059	60	0.27
Total		605	2.76	1622	7.40

#### Clinical manifestations in female chromosomal polymorphism group and control group ( Table 3 and Table 4)

**Table 3:** Clinical manifestations in the female chromosomal polymorphism group and the control group

Type of polymorphism	Total cases	Different chromosome karyotypes	Number of examples	Spontaneous abortion of spouse	Recurrent miscarriage	Fetal abnormality	Primary infertility	Obstructive azoospermia	Non-obstructive azoospermia	Testicular dysplasia	Hypogonadism	Abnormal sperm DNA fragmentation index
qh +	287			40	26	9	107	3	0	10	1	10
		1qh +	165	20	13	3	61	2	0	7	1	8
		9qh +	63	10	9	2	23	0	0	1	0	2
		16qh +	59	10	4	2	23	1	0	2	0	0
Y chromosome polymorphism	1082			136	42	20	287	5	1	18	7	27
		Yqh +	555	73	34	8	195	3	0	13	4	21
		Yqh−	526	62	8	12	92	2	1	5	3	6
		invY	1	1	0	0	0	0	0	0	0	0
qh-	8			1	1	0	4	0	0	0	0	1
pstk +	56			5	1	2	16	0	0	3	0	2
cenh +	13			1	1	0	6	0	1	1	1	0
pss	14			4	0	0	5	0	0	2	0	0
ps−	9			1	0	0	4	0	0	0	0	1
ps +	7			0	1	0	2	0	0	0	0	0
Inv(9)	86			10	6	0	28	0	1	2	1	0
complex polymorphisms	60			13	4	1	33	1	0	1	0	2
total polymorphisms	1622			211	82	32	492	9	3	37	10	43
control group	1630	46, XY		14	8	4	68	2	0	15	6	18

**Table 4:** Comparison of clinical manifestations in the female chromosomal polymorphism group and control group. (control VS polymorphism, ^*^P < 0.05)

Clinical manifestation	Frequency of polymorphic group (n = 605)	Frequency of polymorphic group %	Control group frequency (n = 615)	Frequency of control group %
History of spontaneous abortion	180	29.75	114^*^	18.54^*^
History of recurrent miscarriage	53	8.76	25^*^	4.07^*^
Fetal abnormality	8	1.32	5^*^	0.81^*^
Primary infertility	61	10.08	25^*^	4.07^*^
Uterine abnormality	8	1.32	5^*^	0.81^*^

Amongst the 605 female carriers of chromosomal polymorphism, 180 cases (29.75%) reported a history of spontaneous abortion, with 53 cases (8.76%) experiencing recurrent miscarriages, while the data were 114 cases (18.54%) and 25 cases (4.07%) respectively in the control group (Table 4, *P* < 0.05). A higher incidence rate of fetal abnormalities, Primary infertility, and uterine abnormalities were observed in the chromosomal polymorphism group (Table 4, *P* < 0.05) compared with the control group.

### Clinical presentation in the male chromosomal polymorphism group and the control group ( Table 5 and Table 6)

**Table 5:** Clinical performance of the male chromosomal polymorphism group and the control group in terms of sperm concentration, viability, and sperm morphology

polymorphic type (math.)	Total cases	Different chromosome karyotypes	Number of examples	1 spontaneous abortion	Recurrent miscarriage	Fetal abnormality	Primary infertility	Uterine abnormality
qh +	330			59	31	3	34	3
		1qh +	179	31	15	3	23	2
		9qh +	76	14	9	0	4	1
		16qh +	75	14	7	0	7	0
qh−	3			2	1	0	0	0
pstk +	96			26	6	1	8	2
Cenh +	25			13	1	1	2	1
pss	13			6	4	0	0	1
ps−	9			1	2	0	2	0
ps +	8			1	1	0	1	0
Inv(9)	82			15	7	0	14	1
composite polymorphism	39			4	0	0	0	0
Total polymorphisms	605			127	53	8	61	8
control Group	615	46, XX		89	25	5	25	5

**Table 6:** Clinical manifestations of the male chromosomal polymorphism group and the control group in other respects

Type of polymorphism	Total cases	Different chromosome karyotypes	Number of examples	Mild oligospermia	Moderate oligospermia	Severe oligozoospermia	Extreme oligozoospermia	Mild hypospermia	Moderate weak spermatozoa	Severe hypospermia	Mild Teratozoospermia	Moderate Teratozoospermia	Severe Teratozoospermia
qh +	287			2	2	0	3	23	10	4	10	30	5
		1qh +	165	1	2		2	15	9	3	8	19	2
		9qh +	63	0	0	0	0	4	0	0	2	8	0
		16qh +	59	1	0	0	1	4	1	1	0	3	3
Y chromosome polymorphism	1082			18	24	9	13	96	46	33	49	130	43
		Yqh +	555	9	13	5	3	48	27	14	29	81	15
		Yqh−	526	9	11	4	10	48	19	19	19	49	28
		invY	1	0	0	0	0	0	0	0	1	0	0
qh−	8			0	0	0	0	1	0	2	0	1	1
pstk +	56			1	1	1	0	2	2	3	1	6	0
Cenh +	13			0	1	0	0	1	0	0	0	3	1
pss	14			0	0	0	0	2	1	0	1	1	1
ps−	9			0	0	0	0	0	2	0	1	1	0
ps +	7			0	0	0	0	0	0	0	0	1	0
Inv(9)	86			1	3	1	0	10	4	1	3	11	2
Complex polymorphisms	60			0	0	0	0	4	2	3	6	10	3
Total polymorphisms	1622			22	31	11	16	130	67	46	71	194	56
Control group	1630	46, XY		14	17	8	17	26	33	28	9	38	29

#### Comparison of clinical manifestations in the male chromosomal polymorphism group and the control group ( Table 7)

**Table 7:** Comparison of clinical manifestations in the male chromosomal polymorphism group and the control group. (control vs. polymorphic, ^*^P < 0.01, control vs. polymorphic,^■^*P* < 0.05)

Clinical manifestation	Group frequency of polymorphism (n = 1622)	Polymorphic group frequency %	Control group frequency (n = 1630)	Frequency of control group %
Spouse has a history of spontaneous abortion	371	22.87	169^*^	10.37^*^
Spouse has a history of recurrent miscarriage	98	6.04	29^*^	1.78^*^
Fetal abnormality	32	1.97	4^*^	0.25^*^
Primary infertility	492	30.33	68^*^	4.17^*^
Abnormal sperm quality	667	41.74	117^*^	7.18^*^
Testicular dysplasia	37	2.28	15^■^	0.92^■^
Hypogonadism	10	0.62	6^■^	0.37^■^

Within the cohort of 1622 male polymorphism carriers, 371 cases (22.87%) reported spouses with a history of spontaneous abortion, amongst them, 98 cases (6.04%) experienced recurrent miscarriages. By contrast, amongst the control group of 1630 males, 169 cases (10.37%) had spouses with a history of spontaneous abortion, with 29 cases (1.78%) reporting recurrent miscarriages. We could see a significant difference between these two groups (Table 7, *P* < 0.05).

A total of 32 cases (1.97%) with fetal abnormalities were identified in the polymorphism group, encompassing a spectrum of conditions: congenital heart disease (4 cases), right external ear abnormality (1 case), lymphatic cyst (1 case), NT abnormality (1 case), anencephaly (2 cases), chromosomal abnormalities (9 cases), hydatidiform mole (1 case), facial deformity (1 case), duodenal obstruction (1 case), brain underdevelopment (1 case), common bile duct cyst (1 case), bilateral upper limb deformity (1 case), cleft lip and palate (2 cases), clubfoot and renal dysplasia (1 case), brain tumor (1 case), glioblastoma (1 case), delayed intellectual development (1 case), hand and foot digit deformity (1 case), and hydrocephalus (1 case). The control group reported 4 cases (0.25%) of fetal abnormalities: congenital heart disease (2 cases), cleft lip and palate (1 case), and ear deformity (1 case).

In the polymorphism group, primary infertility was observed in 492 cases (30.33%), which was significantly higher than the 68 cases (4.17%) reported in the control group (Table 7, *P* < 0.05). Additionally, 667 individuals from the polymorphism group (41.74%) exhibited sperm quality issues. These issues were divided into various categories: oligozoospermia (80 cases)—mild (22 cases), moderate (31 cases), severe (11 cases), and extreme (16 cases); asthenozoospermia (243 cases), mild (130 cases), moderate (67 cases), and severe (46 cases); and teratozoospermia (321 cases)—mild (71 cases), moderate (194 cases), and severe (56 cases); obstructive azoospermia (9 cases); non-obstructive azoospermia (3 cases); testicular dysgenesis (37 cases); gonadal hypofunction (10 cases); and abnormal sperm DNA fragmentation index (43). In the control group, sperm quality issues were reported in 117 individuals (7.18%), with 15 cases of testicular dysgenesis, 6 cases of gonadal hypofunction, and 18 cases of abnormal sperm DNA fragmentation index. We also observed a significant difference between these two groups on sperm quality issues (Table 7, *P* < 0.05).

## Discussion

### Elevated detection rate of chromosomal polymorphism in infertile population compared with the general population

Chromosomal polymorphism variations may play a negative role in the occurrence of infertility history, although carriers with chromosomal polymorphism may not necessarily experience reproductive difficulties. Our analysis resulted in a markedly elevated detection rate of 10.16%, which was significantly higher than the chromosomal polymorphism detection rate of approximately 1.77% within the general population [11]. Based on our current data, compared with the normal population, infertile parents who choose assisted reproductive technology may have more diverse chromosomal variations. This result may be related to factors such as the environment, genetics, and lifestyle habits of people in different regions. Our data sources were mainly concentrated in Shandong Province, which was our main source of patients. The manifestation of regional chromosomal polymorphism may require larger data support, which was also the limitation of this study.

### Discrepancies in chromosomal polymorphism detection rates and distribution across genders

Literature suggests a higher prevalence of chromosomal polymorphism among male infertility patients compared with their female counterparts [12]. Our data indicated a 2.7-fold higher detection rate in males than in females. A possible explanation is that female chromosomal mosaicism is limited to somatic cells and usually does not affect the female reproductive axis [13], whereas the incidence of X chromosome abnormalities such as Turner syndrome is low. Thus, the detection rate of male chromosomal abnormalities is higher than that of females. The most common polymorphic karyotypes amongst female and male carriers were qh + and Y qh + , respectively, with qh− and inv (Y) being the least common. The human Y chromosome is highly susceptible to morphological changes [14]. Y qh + refers to an increase in the length of the Y chromosome’s long arm heterochromatin region, which contains genes related to sperm differentiation and development. Excessive duplication of this region can affect chromosome pairing during meiosis, thereby affecting sperm fertilization ability or generation [15]. Notably, 41.74% of individuals exhibited sperm quality issues in our study, this high percentage may be one of the reasons for male infertility, or recurrent miscarriage, or fetal malformations in their wives [14]. Several studies suggested that chromosomal polymorphism can lead to impaired semen quality [13] and an increased risk of infertility in men. Currently, clinical practice suggests that chromosome 9 polymorphism is not an indication of PGT. However, an increasing number of studies suggest that chromosome 9 polymorphism may have a negative impact on normal sperm morphology. Complementary studies underline the predominance of Y chromosome polymorphism in male infertility, alongside a significantly elevated incidence of severe oligozoospermia and azoospermia amongst chromosomal polymorphism carriers compared with individuals with normal karyotypes [16].

### Varied clinical implications of different chromosomal polymorphisms may stem from diverse mechanisms affecting reproductive function.

Chromosomal polymorphism has a certain influence on recurrent miscarriage [17]. Chromosomal polymorphisms primarily manifest in heterochromatic regions, telomeres, centromeres, subcentromeres, and the Y chromosome’s long arm [6, 18]. Despite heterochromatin’s transcriptional inactivity, it crucially mediates sister chromatid cohesion, homologous chromosome pairing, and chromosome segregation [19]. Certain polymorphic variants like pericentric inversions of chromosome 9 engender inversion loops during pairing because of chromatin positional rearrangement [20]. Human chromosome 9 exhibits high polymorphism, with the highest frequency occurring at 9 qh + , followed by inv (9), which may be related to infertility [5]. Inv (9) is one of the common mutations that may interfere with early embryo implantation and endometrial receptivity [21], and the specific molecular mechanism needs to be verified through scientific molecular biology experiments. This result may induce heterochromatinisation in adjacent euchromatic regions and specific gene microdeletions [22], culminating in unbalanced gamete formation during meiosis, aneuploid embryo generation, and ensuing early miscarriages [23, 24].

### Chromosomal polymorphism carriers exhibit a high prevalence of reproductive abnormalities

The majority of male factors leading to infertility are idiopathic azoospermia or severe oligozoospermia. Chromosomal polymorphism, to a certain extent, leads to spermatogenesis disorders and is one of the important genetic factors in male infertility patients. A negative correlation exists between male chromosomal polymorphism and fertilization, cleavage, and high-quality embryo formation in assisted reproduction [25]. The emergence of ICSI technology can improve the clinical outcomes of male patients with chromosomal polymorphisms [26], as chromosomal polymorphism is an important factor affecting fertilization in some patients. Many factors affect female fertility, such as immunity, anatomy, endocrine system, and genetics. Chromosomal polymorphism variation is an important factor leading to female infertility. Our data consistently demonstrate a noteworthy correlation between chromosomal polymorphism and a propensity for recurrent miscarriages. Our study identified a correlation between chromosomal polymorphism and fetal malformations, although the specific types of malformations displayed no discernible pattern. The influence of chromosomal polymorphism carriers on their fertility remains a subject of ongoing debate. Nevertheless, the variations in chromosomal polymorphism must not be overlooked, and further comprehensive research is warranted to elucidate the mechanisms underlying its impact on pregnancy outcomes.

## Conclusion

Chromosomal polymorphism may have a certain negative correlation with reproductive abnormalities, including spontaneous abortion, fetal anomalies, and deterioration in male sperm quality, further clarification of chromosomal polymorphism is crucial for the diagnosis, treatment, and assisted reproduction of infertile patients.

## Data Availability

No datasets were generated or analysed during the current study.
